# Enhancement of Charge Separation and NIR Light Harvesting through Construction of 2D–2D Bi_4_O_5_I_2_/BiOBr:Yb^3+^, Er^3+^ Z‐Scheme Heterojunctions for Improved Full‐Spectrum Photocatalytic Performance

**DOI:** 10.1002/advs.202207514

**Published:** 2023-02-21

**Authors:** Yongjin Li, Junhao Ma, Liang Xu, Tong Liu, Taizhong Xiao, Daomei Chen, Zhiguo Song, Jianbei Qiu, Yueli Zhang

**Affiliations:** ^1^ School of Materials Science and Engineering Kunming University of Science and Technology Kunming 650093 P. R. China; ^2^ State Key Laboratory of Optoelectronic Materials and Technologies School of Materials Science and Engineering Sun Yat‐Sen University Guangzhou 510275 P. R. China; ^3^ National Center for International Research on Photoelectric and Energy Materials School of Materials and Energy Yunnan University Kunming 650091 P. R. China

**Keywords:** Bi_4_O_5_I_2_/BiOBr:Yb^3+^,Er^3+^, full spectrum, photocatalytic performance, upconversion, Z‐scheme heterojunctions

## Abstract

Developing full‐spectrum photocatalysts with simultaneous broadband light absorption, excellent charge separation, and high redox capabilities is becoming increasingly significant. Herein, inspired by the similarities in crystalline structures and compositions, a unique 2D–2D Bi_4_O_5_I_2_/BiOBr:Yb^3+^,Er^3+^ (BI‐BYE) Z‐scheme heterojunction with upconversion (UC) functionality is successfully designed and fabricated. The co‐doped Yb^3+^ and Er^3+^ harvest near‐infrared (NIR) light and then convert it into visible light via the UC function, expanding the optical response range of the photocatalytic system. The intimate 2D–2D interface contact provides more charge migration channels and enhances the Förster resonant energy transfer of BI‐BYE, leading to significantly improved NIR light utilization efficiency. Density functional theory (DFT) calculations and experimental results confirm that the Z‐scheme heterojunction is formed and that this heterojunction endows the BI‐BYE heterostructure with high charge separation and strong redox capability. Benefit from these synergies, the optimized 75BI‐25BYE heterostructure exhibits the highest photocatalytic performance for Bisphenol A (BPA) degradation under full‐spectrum and NIR light irradiation, outperforming BYE by 6.0 and 5.3 times, respectively. This work paves an effective approach for designing highly efficient full‐spectrum responsive Z‐scheme heterojunction photocatalysts with UC function.

## Graphical abstract

A 2D–2D Bi_4_O_5_I_2_/BiOBr:Yb^3+^,Er^3+^ Z‐scheme heterojunction with upconversion (UC) functionality is successfully designed and fabricated. It improves its full‐spectrum photocatalytic activity due to the synergistic effects from UC function, 2D–2D heterointerface, and Z‐scheme charge transfer process. This work paves an effective approach for designing highly efficient full‐spectrum responsive Z‐scheme heterojunction photocatalysts with UC function.

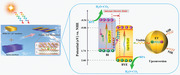

## 1 Introduction

Photocatalytic technology is a promising route for the development and utilization of solar energy, and it is regarded as a win‐win strategy for addressing environmental pollution and the energy crisis.^[^ ^1^, ^2^, ^3^, ^4^, ^5^ ^]^ In practical applications, the maximum utilization of the full solar spectrum is crucial for improving the photocatalytic efficiency of photocatalysts. However, most semiconductor photocatalysts are only active under ultraviolet (UV) light and some of the visible (Vis) light spectrum. Near‐infrared (NIR) light, which represents nearly 50% of solar light, is rarely harvested, which limits the practical application of semiconductor photocatalysts.^[^ ^6^, ^7^, ^8^ ^]^ In addition, compared with UV and Vis light, the NIR light has a strong penetrating capacity, which is of benefit for sufficient contact with substrates and solid–liquid photocatalytic in the photocatalytic process.^[^ ^9^ ^]^ Thus, developing NIR light‐responsive photocatalyst is a very urgent task. To efficiently utilize NIR light, lanthanide (Ln^3+^)‐doped upconversion (UC) materials have been investigated as an effective strategy because these materials have the capability of converting NIR radiation into UV and visible light.^[^ ^10^, ^11^, ^12^, ^13^, ^14^, ^15^ ^]^ Unfortunately, the photocatalytic activity of these UC materials is relatively low because their utilization efficiency of UC photon energy is not very high. Furthermore, the rapid recombination of carriers is also an issue. Therefore, designing novel and efficient photocatalysts with the full‐spectrum response and high carrier separation efficiency would be highly significant for efficiently utilizing solar energy.

In recent years, the investigation of bismuth oxyhalides has become a research hotspot. These materials show promise as candidates for photocatalysis and energy conversion due to their adjustable solar absorption range and unique layered structure.^[^ ^16^, ^17^, ^18^ ^]^ Therefore, bismuth oxyhalides have been studied for various applications including hydrogen production,^[^ ^19^ ^]^ CO_2_ reduction,^[^ ^20^, ^21^ ^]^ N_2_ fixation,^[^ ^22^, ^23^ ^]^ and especially organic degradation.^[^ ^10^, ^24^, ^25^, ^26^ ^]^ In our previous work, we discovered that Yb^3+^/Er^3+^ doped BiOBr nanosheets can effectively harvest green UC light to produce electron–hole pairs for the photodegradation of Rhodamine B (RhB) and Bisphenol A (BPA) by utilizing NIR light.^[^ ^27^, ^28^, ^29^ ^]^ However, the overall photocatalytic capacity of these BiOBr:Yb^3+^/Er^3+^ nanosheets was not satisfactory due to their low UC conversion efficiency and limited visible light absorption performance. Strategies such as doping,^[^ ^30^ ^]^ vacancy engineering,^[^ ^29^ ^]^ or constructing heterostructures^[^ ^31^, ^32^, ^33^ ^]^ have been applied to improve the UC luminescence intensity and extend the light absorption range of bismuth oxyhalides. Therefore, enhancing the photocatalytic efficiency of these materials is highly significant for developing bismuth‐based catalysts with full‐spectrum response.

The construction of Z‐scheme heterojunctions has proven to be an effective strategy for boosting photocatalytic efficiency.^[^ ^34^ ^]^ These heterojunctions can simultaneously enhance the light absorption ability of photocatalysts, facilitate charge separation, and lead to high redox potential.^[^ ^35^ ^]^ In general, the interfacial facets and proper band structure are two important factors that influence the photocatalytic performance of semiconductor heterostructures. Considering the 2D structure of BiOBr, investigating the combination of another 2D material with BiOBr shows good promise. This is because 2D–2D heterojunctions possess large contact surfaces, abundant active sites, and a short carrier migration distance.^[^ ^36^, ^37^, ^38^, ^39^ ^]^ These factors are beneficial for improving the separation and transfer of charges across heterojunction interfaces. In addition, strong interactions and electronic coupling are also advantageous for improving photocatalytic activity. To date, some researchers have designed and constructed 2D–2D heterojunctions using 2D BiOBr. These include CoS/BiOBr,^[^ ^40^ ^]^ CoAl‐LDH/BiOBr,^[^ ^41^ ^]^ WO_3_/BiOBr,^[^ ^42^ ^]^ 2D–2D heterojunctions, which demonstrate greatly enhanced photocatalytic performance.

In addition to a suitable interfacial configuration, a matched band structure is required for the construction of high‐efficiency 2D–2D Z‐scheme heterojunctions. Similar to BiOBr, Bi_4_O_5_I_2_ has a typical 2D layered structure. Bi_4_O_5_I_2_ also exhibits excellent visible light capture properties, higher conduction band position, and strong reduction ability.^[^ ^43^ ^]^ According to previous reports,^[^ ^44^ ^]^ the conduction band (CB) position and valence band (VB) positions of Bi_4_O_5_I_2_ are around −1.02 and 1.05 eV versus a normal hydrogen electrode (NHE), respectively. Moreover, the VB and CB potentials for BiOBr are obviously lower than that of Bi_4_O_5_I_2_, respectively, suggesting that the photogenerated electrons in the CB of BiOBr could transfer to the VB of Bi_4_O_5_I_2_ following a Z‐scheme mechanism when they combine. More importantly, in view of their similar structures and the composition of Bi_4_O_5_I_2_, this material is expected to form a close interfacial contact with BiOBr. Ln^3+^ ion doping is an additional strategy that can enable the use of BiOBr and Bi_4_O_5_I_2_ in UC‐based applications.^[^ ^45^, ^46^ ^]^ Therefore, Ln^3+^ doping is expected to provide a material with simultaneous UC functionality, a Z‐scheme heterojunction, and a 2D–2D heterojunction interface. This strategy is expected to improve photocatalytic performance. Nevertheless, to the best of our knowledge, few research reports have been published regarding the construction of 2D–2D Bi_4_O_5_I_2_/BiOBr:Yb^3+^,Er^3+^ Z‐scheme heterojunctions, and the underlying mechanism of this material is still unclear.

Inspired by the unique features discussed above, for the first time, we designed and fabricated 2D material‐based Bi_4_O_5_I_2_/BiOBr:Yb^3+^,Er^3+^ Z‐scheme heterojunctions that constructively coupled the UC function of lanthanide ions (Ln^3+^) and a heterojunction to simultaneously achieve a broad light response range, high redox capability, and efficient charge separation. These Bi_4_O_5_I_2_/BiOBr:Yb^3+^,Er^3+^ heterojunctions exhibited superior photocatalytic activity for the degradation of BPA under the irradiation of both NIR and full‐spectrum light. Furthermore, the possible photocatalytic mechanism of Bi_4_O_5_I_2_/BiOBr:Yb^3+^,Er^3+^ heterojunctions was systematically investigated.

## 2 Results and Discussion

### 2.1 Characterization of Materials

**Scheme 1:**
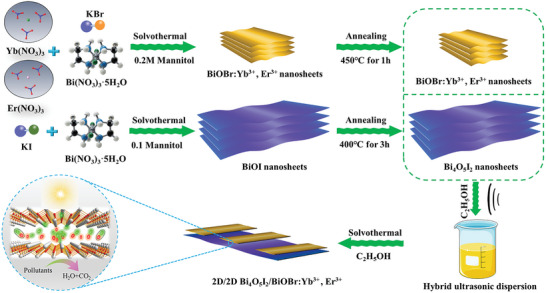
Schematic illustration of the synthesis of 2D–2D Bi_4_O_5_I_2_/BiOBr:Yb^3+^,Er^3+^ Z‐scheme heterojunctions.

The 2D–2D Bi_4_O_5_I_2_/BiOBr:Yb^3+^,Er^3+^ Z‐scheme heterojunctions were prepared by a two‐step solvothermal method, as presented in **Scheme** **1**. The prepared samples were denoted as (100‐*x*)Bi_4_O_5_I_2_‐*x*BiOBr:Yb^3+^,Er^3+^, where *x* refers to the percentage of BiOBr:Yb^3+^,Er^3+^ (*x* = 0, 25, 50, 75, 100). These were abbreviated as BI, 75BI‐25BYE, 50BI‐50BYE, 25BI‐75BYE, and BYE. Figure S1 (Supporting Information) shows the zeta potentials of the as‐prepared BYE and BI in ethanol solvent. The BYE surface was negatively charged with a zeta potential of −36.1 mV, whereas the BI surface was positively charged with a zeta potential of 22.9 mV. These oppositely charged zeta potentials resulted in strong electrostatic attraction between BYE and BI, which was beneficial for increased contact and charge transfer between these two components.^[^ ^38^ ^]^ This led to spontaneous assembly and formation of a close heterointerface contact. Thus, a 2D–2D BI‐BYE heterojunction was obtained through a Coulomb electrostatic self‐assembly strategy.

**Figure 1:**
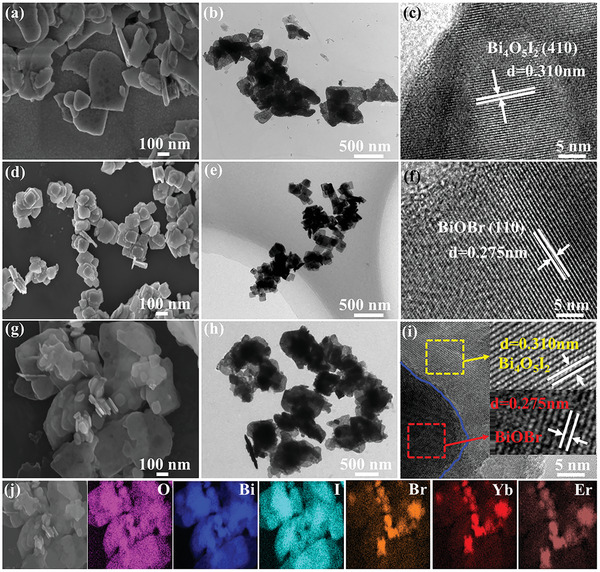
a) SEM, b) TEM, and c) HRTEM image of BI; d) SEM, e) TEM and f) HRTEM image of BYE; g) SEM, h) TEM, and i) HRTEM image of 75BI‐25BYE; j) EDS elemental mapping images of 75BI‐25BYE heterojunctions.

The morphologies and microstructures of the as‐prepared BI, BYE, and 75BI‐25BYE were observed using scanning electron microscopy (SEM) and transmission electron microscopy (TEM). BI displayed irregular 2D large‐scale sheet‐shaped structures with a particle size of 500–1000 nm (**Figure** **1**a). 2D nanosheet morphologies show good promise for combination in a heterojunction with other semiconductor materials.^[^ ^47^ ^]^ TEM analysis further revealed this sheet‐shaped structure (Figure 1b). The high‐resolution TEM (HRTEM) image of BI showed a clear lattice fringe spacing of 0.310 nm, which was assigned to the Bi_4_O_5_I_2_ (410) crystal plane (Figure 1c). Meanwhile, the prepared BYE also exhibited a 2D sheet morphology, but it showed a smaller particle size of about 100 nm (Figure 1d,e). HRTEM analysis (Figure 1f) showed a distinct lattice spacing of 0.275 nm, which was ascribed to the BiOBr (110) plane. This was consistent with the result of pure BiOBr nanosheets (Figure S2, Supporting Information), indicating the doping Yb^3+^ and Er^3+^ ions had little effect on lattice fringes of BiOBr. The SEM image of the 75BI‐25BYE heterojunction showed that small BYE particles were tightly anchored on a large BI structure (Figure 1g). The TEM image of the 75BI‐25BYE heterojunction further revealed that small nanosheets were closely stacked on the surface of the larger sheet (Figure 1h). HRTEM analysis of this heterojunction showed the existence of an obvious interface between BI and BYE (denoted by the blue line, Figure 1i and Figure S3, Supporting Information) and the presence of a clear lattice fringe. The lattice distance of 0.310 nm was in good agreement with the (410) plane of Bi_4_O_5_I_2_, whereas the lattice fringe of 0.275 nm was assigned to the (110) plane of BiOBr. The identical Bi and O atoms created the conditions for lattice matching, facilitating the formation of covalent bonds and leading to the construction of a strong interfacial connection. In addition, energy dispersive X‐ray spectroscopy (EDX) elemental mapping analysis (Figure 1j) clearly showed the characteristic Bi, O, I, Br, Yb, and Er elements in the 75BI‐25BYE heterojunction. The Bi, O, and I elements were homogeneously distributed across the whole nanosheet, but the Br, Yb, and Er elements were localized in several small areas, indicating that the BYE nanosheets were dispersed on the surface of the BI nanoplate. This was in good agreement with the SEM and TEM images. These results collaboratively reveal the formation of an intimate 2D–2D interaction in the composite.

**Figure 2:**
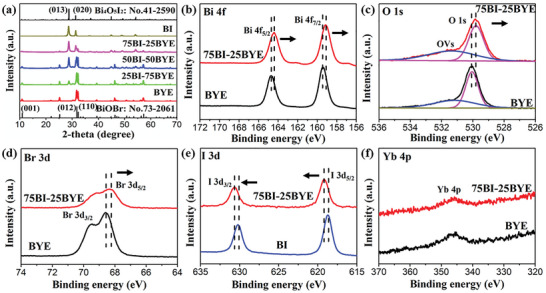
a) XRD patterns of BI‐BYE heterojunctions; XPS spectra of BI‐BYE heterojunctions, b) Bi 4f; c) O 1s; d) I 3d; e) Br 3d; f) Yb 4p.

The phase compositions and crystal structures of the as‐synthesized catalysts were investigated by X‐ray diffraction (XRD) measurements (Figure S4, Supporting Information and **Figure** **2**a). BYE and BI showed diffraction patterns consistent with those of tetragonal BiOBr (JCPDS No.73‐2061) and monoclinic Bi_4_O_5_I_2_ (JCPDS No.41‐2950), respectively. The diffraction peaks at 2*θ* = 10.86°, 25.19°, 31.71°, 32.25°, 46.24°, and 57.14° corresponded to the (001), (011), (012), (110), (020), and (212) planes of BiOBr. The diffraction peaks at 2*θ* = 28.68°, 31.36°, and 54.52° represented the (013), (402), and (‐715) planes of Bi_4_O_5_I_2_, respectively. As expected, the prepared BI‐BYE heterojunctions displayed the characteristic peaks of both BYE and BI, and no impurities were detected. However, the intensity of the (013) diffraction peak gradually increased with increasing proportion of BI in the heterojunction (Figure S5, Supporting Information), which further demonstrated that the heterojunctions were successfully prepared.

To further investigate the chemical state and interface interaction in the BI‐BYE heterojunctions, X‐ray photoelectron spectroscopy (XPS) was performed. The XPS survey spectrum (Figure S6, Supporting Information) revealed the presence of Bi, O, Br, and I in the composite heterojunction, which was in good agreement with the EDS test results. The high‐resolution Bi 4f XPS spectrum of BYE (Figure 2b) showed two peaks at 159.1 and 164.4 eV that were respectively ascribed to Bi 4f_7/2_ and Bi 4f_5/2_ of Bi^3+.[^ ^29^ ^]^ In comparison, the Bi 4f peak of 75BI‐25BYE was shifted to a lower binding energy. The O 1s peak of BYE was deconvoluted into two peaks at 529.8 and 531.4 eV, which corresponded to lattice oxygen (Bi^3+^‐O) and the oxygen atoms near the oxygen vacancies (OVs), respectively.^[^ ^22^ ^]^ The higher concentration of OVs and the shift in the lower binding energy direction of the lattice oxygen peak in the 75BI‐25BYE O 1s spectrum indicated that more dangling oxygen atoms were generated near the vacancies.^[^ ^48^ ^]^ The existence of OVs on 75BI‐25BYE was directly demonstrated by electron paramagnetic resonance (EPR) spectroscopy. In Figure S7 (Supporting Information), the peak observed at *g* = 2.002 is a typical signal of OVs,^[^ ^29^ ^]^ and the intensity of EPR signal for 75BI‐25BYE is higher than that of BYE, indicating a higher concentration of OVs in 75BI‐25BYE.OVs are the important active site for O_2_ adsorption and activation. Thus, the OVs formed on 75BI‐25BYE can improve the O_2_ conversion to •O^2−,[^ ^29^ ^]^ dramatically affecting photocatalytic performance. Two splitting peaks around 68.5 and 69.5 eV were observed in the Br 3d spectrum of BYE (Figure 2e), which were ascribed to the 3d_5/2_ and 3d_3/2_ signals of Br^−.[^ ^28^ ^]^ After the incorporation with BI, the 75BI‐25BYE Br 3d spectrum exhibited a noticeable 0.35 eV shift to a lower binding energy. The binding energy shift of these corresponding peaks can be ascribed to the construction of a strong interfacial interaction between BYE and BI, revealing that the coupling state of the BI‐BYE heterojunction was not just a simple physical attachment. Generally, a negative shift in binding energy indicates an increase in electron density, and vice versa.^[^ ^49^ ^]^ Thus, the electron migration pathway in these composites can be determined from the shift of their XPS binding energies. In this case, the negative shifts of the Bi 4f, O 1s, and Br 3d peaks imply that BYE acted as an electron acceptor in the BI‐BYE heterojunction. To further reveal the electron transportation direction, high‐resolution I 3d spectra of BI and 75BI‐25BYE were also examined. In the I 3d spectrum of BI (Figure 2e), the peaks at 619.1 and 630.6 eV respectively corresponded to the I 3d_5/2_ and I 3d_3/2_ signals of I^−.[^ ^46^ ^]^ A significant 0.41 eV shift to higher binding energies was observed for the I 3d states of 75BI‐25BYE, implying a decline in electron density. In addition, compared with pure Bi_4_O_5_I_2_ and BiOBr (Figure S8, Supporting Information), peaks were observed at 345.5 eV in the BYE and 75BI‐25BYE Yb 4d spectra, suggesting the presence of Yb^3+^. According to these phenomena and previous reports,^[^ ^50^ ^]^ it can be deduced that electron migration occurs from BI to BYE in the BI‐BYE heterojunction. The detailed mechanism of the electron transportation pathway in these 2D–2D BI‐BYE heterojunctions will be further elaborated later.

**Figure 3:**
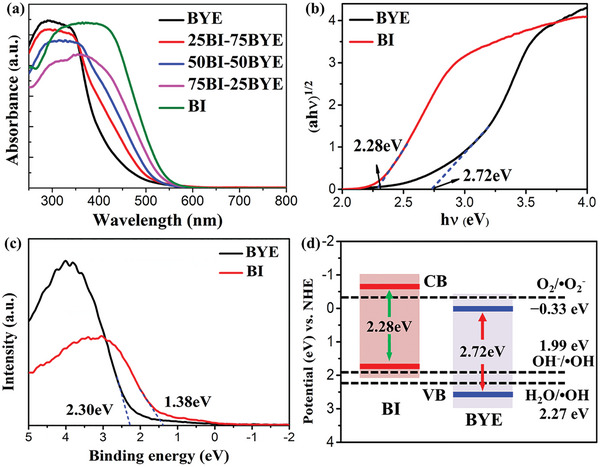
a) Absorption spectra; b) Plots of (*αhv*)^1/2^ versus *hν* of BI‐BYE heterojunctions; c) VB‐XPS of BI and BYE; d) Schematic band structure of BI and BYE.

UV–Vis absorption spectroscopy was used to investigate the optical properties of the samples (**Figure** **3**a). BYE exhibited limited absorption in the visible light range, with an absorption edge of approximately 450 nm. In contrast, BI showed an absorption edge of about 560 nm, indicating its excellent visible light response. Therefore, after the formation of the BI‐BYE heterojunction, the light absorption of the sample expanded to longer wavelengths. The bandgaps (*E*_g_) of BI and BYE were estimated to be 2.28 and 2.72 eV based on the Kubelka‐Munk equation:^[^ ^51^ ^]^ *ahv* = *A*(*hv* − *E_g_*)^*n*^ (Figure 3b). The valence band potentials (*E*_VB, XPS_) of BI and BYE were respectively determined to be 1.38 and 2.30 eV based on their XPS valence band (VB‐XPS) spectra (Figure 3c). The equation *E*_VB, NHE_ = *φ* + *E*_VB, XPS_ − 4.44,^[^ ^50^ ^]^ where *φ* is the work function of the XPS instrument (*φ* = 4.8 eV) and *E*_VB, NHE_ is the VB position versus NHE, was used to determine *E*_VB, NHE_ values. The *E*_VB, NHE_ value of BI was 1.74 eV and that of BYE was 2.66 eV. Moreover, according to the empirical formula: *E*_VB_  =  *E*_CB_ + *E*_g_,^[^ ^52^ ^]^ the *E*_CB_ value of BI was −0.54 eV and that of the BYE was −0.06 eV. The calculated *E*_CB_ of BI and BYE well matched the values of −0.57 and −0.03 eV, respectively, from the Mott‐Schottky plots well (Figure S9, Supporting Information). Based on this analysis, the BI and BYE band structures are illustrated in Figure 3d. These band structures ensure the formation of the Z‐scheme heterojunction between BI and BYE.

### 2.2 Photocatalytic Performance

**Figure 4:**
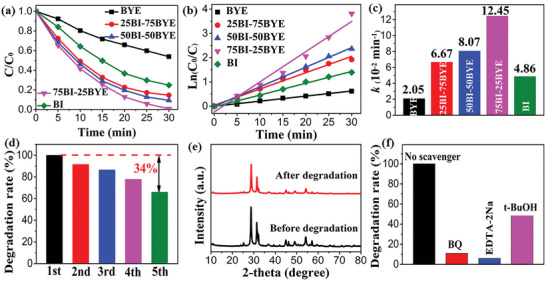
a) Photocatalytic degradation of BPA, b,c) apparent rate constants of BI‐BYE heterojunctions under full‐spectrum light irradiation; d) Cycling experiment, e) XRD pattern of before and after five cycles reaction; f) Trapping experiment for 75BI‐25BYE under full‐spectrum light irradiation.

The typical organic pollutant BPA was selected as the degradation target for evaluating the photocatalytic activity of the as‐prepared BI‐BYE heterojunction. BPA is very stable, and the self‐degradation of BPA barely occurs in the absence of a photocatalyst.^[^ ^30^ ^]^ The photocatalytic BPA degradation rates of BYE and BI were about 47% and 78% after 30 min of full‐spectrum light irradiation (**Figure** **4**a). However, compared with BYE and BI, the BI‐BYE heterojunction demonstrated significantly enhanced BPA degradation performance. In particular, the 75BI‐25BYE heterojunction exhibited the highest BPA degradation efficiency, reaching 100% BPA degradation within 30 min. BPA degradation followed pseudo‐first‐order kinetics^[^ ^30^ ^]^ (Figure 4b,c), and the kinetic constants (*k*) of BYE, 25BI‐75BYE, 50BI‐50BYE, 75BI‐25BYE, and BI were 0.021, 0.067, 0.081, 0.125, and 0.060 min^−1^, respectively. The 75BI‐25BYE heterojunction sample exhibited the highest BPA degradation kinetic constant, with a value 2.0 and 6.0 times higher than those of BI and BYE, respectively. The photocatalytic activity under Vis light irradiations also showed consistent results (Figure S10, Supporting Information). This demonstrates the formation of a heterojunction and the interaction between BYE and BI, which led to a significant enhancement in photocatalytic activity.

Catalyst stability and recoverability are very important factors for their practical application. After five catalytic cycles, 75BI‐25BYE exhibited a BPA degradation rate that was nearly 34% lower than the rate achieved in the first cycle (Figure 4d). In the XRD spectra (Figure 4e), the overall characteristic peaks were retained after five cycles, but the peak intensity decreased. Additionally, the stability of 75BI‐25BYE was analyzed by SEM and XPS, as shown in Figure S11 (Supporting Information). No obvious morphological changes were observed by studying the catalyst before and after the reaction, and the small BYE particles remained tightly anchored on the large BI structure, verifying their favorable stability and reusability. Figure S11b,c (Supporting Information) displays the high‐resolution XPS spectra of Br 3d and O 1s after five cycles. Evidently, the Br 3d and O 1s peaks did not exhibit a perceptible shift. These results indicated the stability of the crystal structure of 75BI‐25BYE. However, the concentration of OVs decreased after the photocatalytic degradation of BPA. A similar phenomenon has also been reported in the literature.^[^ ^29^, ^53^ ^]^ As shown in Figure S11d (Supporting Information), the decreased EPR signals of 75BI‐25BYE after five cycles verify that the OVs were partially consumed, leading to lower photocatalytic activity. Therefore, the gradual decline in the BPA degradation efficiency of 75BI‐25BYE was mainly caused by the loss of the catalyst and reduced OVs.

Reactive species trapping experiments were carried out to obtain a more comprehensive understanding of the enhanced photocatalytic activity mechanism of the 75BI‐25BYE heterojunction for BPA degradation (Figure 4f). Ethylenediaminetetraacetic acid disodium salt (EDTA‐2Na) was utilized as the sacrificial agent for hole (*h* ^+^), Benzoquinone (BQ) was used as the sacrificial agent for superoxide radicals (•O_2_^−^), and Tertiary butanol (t‐BuOH) was used as the sacrificial agent for hydroxyl radical (•OH). The BPA degradation rate sharply decreased after adding EDTA‐2Na or BQ, while it slightly decreased after adding t‐BuOH. These results showed that *h* ^+^, •O_2_^−^, and •OH were active species for the photocatalytic degradation of BPA, and their influence followed the order *h* ^+^ > •O_2_^−^ > •OH.

**Scheme 2:**
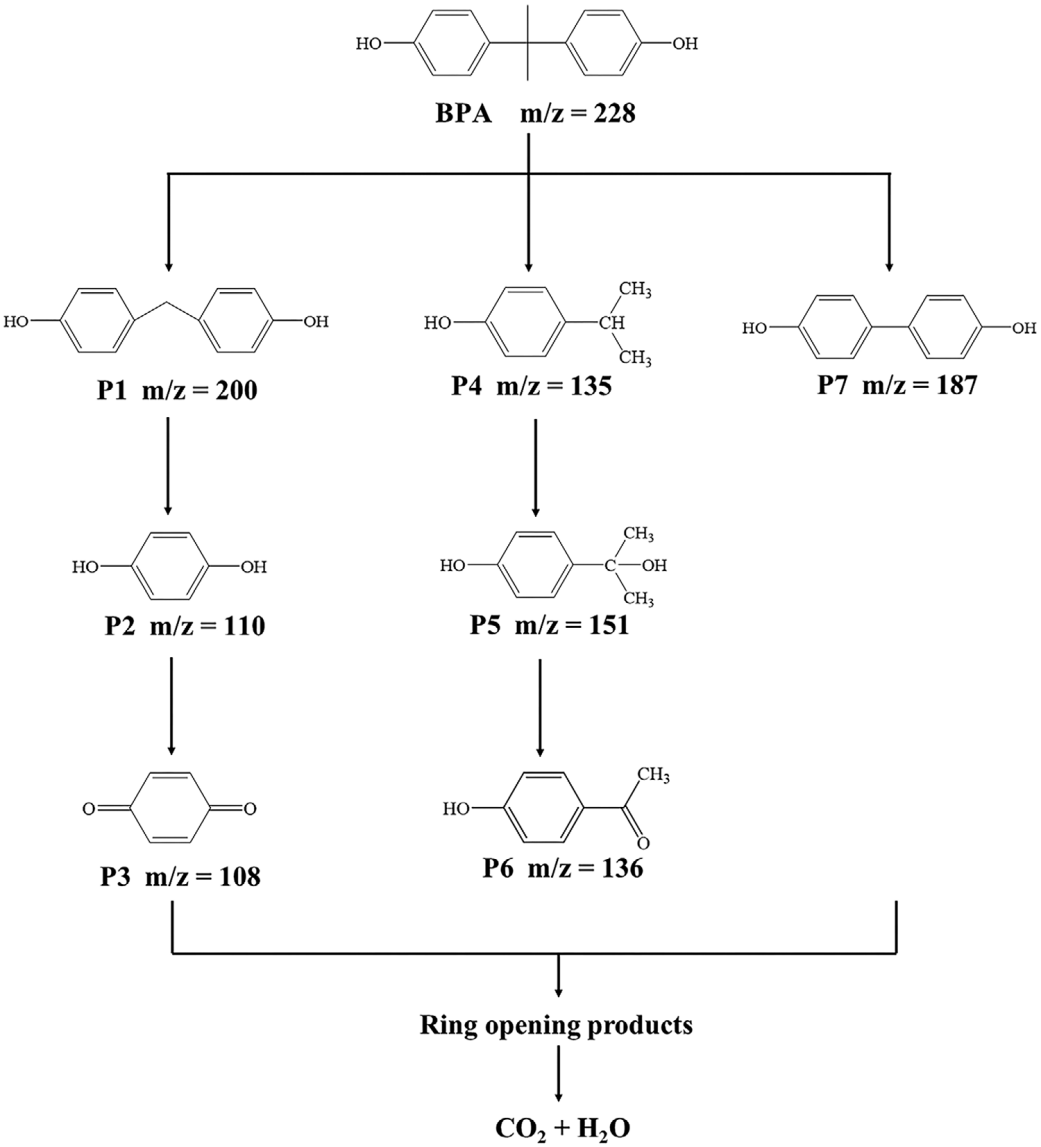
Proposed degradation pathways of BPA in 75BI‐25BYE heterojunction under full‐spectrum light irradiation.

In addition, the measurements of total organic carbon (TOC) were carried to deeply investigate photocatalytic activities (Figure S12, Supporting Information). It was found the TOC removal efficiency (65%) was much lower than the was lower than BPA removal rate (100%), demonstrating some intermediates must be formed during the BPA degradation process.^[^ ^54^ ^]^ To understand the degradation pathways of BPA in the BI‐BYE heterojunction system, the decomposition intermediates of BPA were identified by liquid chromatography‐mass spectrometry (LC‐MS) and shown in Figure S13 (Supporting Information). The detected main intermediates were listed and shown in Table S1 (Supporting Information). On the basis of the experimental results, the possible photocatalytic degradation pathways of BPA over 75BI‐25BYE heterojunction under full‐spectrum light irradiation was proposed and illustrated in **Scheme** **2**. One degradation pathway was that the benzene ring or hydroxyl groups in the BPA molecules were attacked by •OH formed demethylation pathway, resulting in the BPA may be dehydrogenized directly to generate 4,4’‐methylenediphenol (P1, m/z = 200),^[^ ^55^ ^]^ which was further converted into hydroquinone (P2, m/z = 110) via cleavage of the of the C—C bond. Due to the hydroquinone was relatively unstable, which can be further oxidized to 4‐benzoquinone (P3, m/z = 108).^[^ ^56^ ^]^ In another degradation pathway, the C—C single bond between isopropyl and benzene rings would be attacked by •OH, resulting in the formation of 4‐isopropylphenol (P4, m/z = 135) through the *β*‐scission reaction.^[^ ^56^, ^57^, ^58^ ^]^ Then, 4‐isopropylphenol further converted into 4‐isopropanolphenol (P5, m/z = 151) by the hydroxylation pathway. Under the sustained action of •OH, 4‐isopropylphenol was induced to be the 4‐hydroxyacetophenone (P6, m/z = 136) after the carbonylation reaction. In addition, BPA can be directly decomposed to produce 4,4’‐dihydroxybiphenyl (P7, m/z = 187) under the attack of *h* ^+^ through dealkylation reaction.^[^ ^59^ ^]^ Finally, these intermediates were further oxidized to form low molecular ring‐opening products, and even eventually mineralized into CO_2_ and H_2_O, which further supported the TOC removal analysis.

### 2.3 Influencing Factors Affecting Photocatalytic Performance

**Figure 5:**
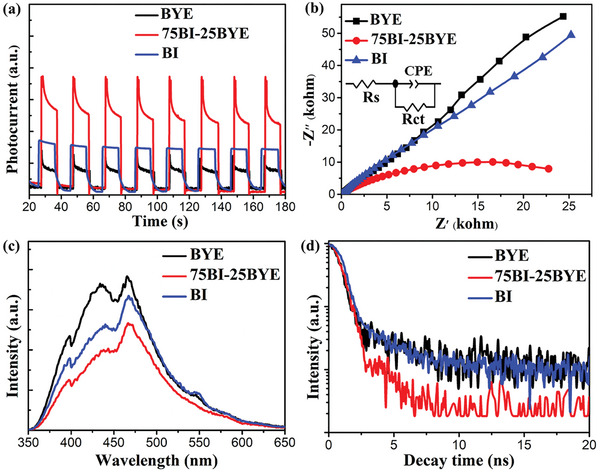
a) Transient photocurrent response; b) EIS; c) PL spectra; d) TRPL spectra of BI‐BYE heterojunctions.

To further investigate the significantly improved photocatalytic performance of the BI‐BYE heterojunction, related photo/electrochemical properties were studied. As depicted in **Figure** **5**a, all prepared samples exhibited a rapid photocurrent response under light irradiation, indicating the formation of photogenerated electron–hole pairs. It can be observed that the photocurrent density of 75BI‐25BYE was much higher than that of BYE and BI, which demonstrated that 75BI‐25BYE possessed excellent separation efficiency of photogenerated charges. This confirmed that the construction of Z‐scheme heterojunction effectively improved the separation of electron–hole pairs. The charge separation and transfer behavior of BI‐BYE heterojunction was further determined by electron impedance spectroscopy (EIS). Popularly, the smaller semicircle arc of EIS Nyquist plots represented the lower interfacial transfer resistance and the faster the process of interfacial carrier transfer.^[^ ^60^ ^]^ As expected, 75BI‐25BYE exhibited the smallest arc radius, as delineated in Figure 5b, indicating lower charge transfer resistance and faster interfacial charge transfer rate in 75BI‐25BYE, which was consistent with the photocurrent test results. In order to acquire the quantitative result, the fitted values of impedance were presented in Table S2 (Supporting Information), and the corresponding simulated equivalent circuit was inserted in Figure 5b. Obviously, the charge transfer resistance (*R*_ct_) of 75BI‐25BYE (31.76 kΩ) was significantly decreased than that of BYE (875.8 kΩ) and BI (587.4 kΩ), manifesting that the construction of BI‐BYE heterojunction can decrease the interfacial charge transfer resistance, which is in favor of photogenerated carriers transfer and separation. To better research the recombination rate of electron–hole pairs, the photoluminescence (PL) spectra were measured. Steady‐state PL spectra showed that 75BI‐25BYE exhibited the lowest PL intensity compared with BYE and BI (Figure 5c), suggesting that the formation of the heterojunction effectively suppressed the photogenerated electron‐hole pair recombination. Time‐resolved PL (TRPL) decay spectra were obtained to study the dynamics of the photogenerated charge carriers. As exhibited in Figure 5d, the fluorescence attenuation curve was fitted using a double‐exponential function:^[^ ^50^ ^]^

(1) $$I\left(t\right)=A_{1}\exp\left(-t/\tau_{1}\right)+A_{2}\exp\left(-t/\tau_{2}\right)$$

where *τ*_1_ and *τ*_2_ are the emission lifetimes, and *A*_1_ and *A*_2_ are the corresponding amplitudes. Meanwhile, the average decay lifetimes (*τ*_A_) are defined as:

(2) $$\tau_{A}=\left(A_{1}\tau_{1}^{2}+A_{2}\tau_{2}^{2}\right)/\left(A_{1}\tau_{1}+A_{2}\tau_{2}\right)$$

As listed in **Table** **1**, the short lifetime component *τ*_1_ originated from the nonradiative recombination of charge carriers in the surface defect states, while the longer lifetime component of *τ*_2_ was caused by free exciton recombination in BYE. It can be clearly seen that the 75BI‐25BYE shows a shorter average lifetime (1.26 ns) than that of BYE (1.58 ns) and BI (1.55 ns). According to the literature,^[^ ^61^, ^62^, ^63^, ^64^ ^]^ a reduced decay lifetime suggests that more effective charge carrier formation and faster interfacial charge transfer occur in the heterojunction structure due to the emergence of a nonradiative energy transfer process. Thus, this phenomenon implies that the defect states caused by OVs on 75BI‐25BYE provide extra non‐radiative pathways for the transport of charge carriers, which is accountable for the decreased fluorescence lifetime. The reduced decay lifetime and quenched PL suggest the more effective charge carrier formation and faster interfacial charge transfer occurring in the BI‐BYE heterojunction. These photo/electrochemical results demonstrate that the construction of the BI‐BYE heterojunction led to the severe inhibition of photogenerated electron–hole pair recombination and effectively improved the separation and transport of the photogenerated charge carriers. As a result, the photocatalytic activity of the heterojunction was enhanced compared to that of the separate BYE and BI nanosheets.

**Table 1:** Kinetic parameters of the fitting decay parameters of BYE and 75BI‐25BYE heterojunction

Samples	*τ*_1_ [ns]	*A*_1_[%]	*τ*_2_ [ns]	*A*_2_[%]	*τ*_A_ [ns]
BYE	0.56	74.75	4.63	25.25	1.58
75BI‐25BYE	0.54	78.33	3.87	21.67	1.26
BI	0.55	72.52	4.19	27.48	1.55

**Figure 6:**
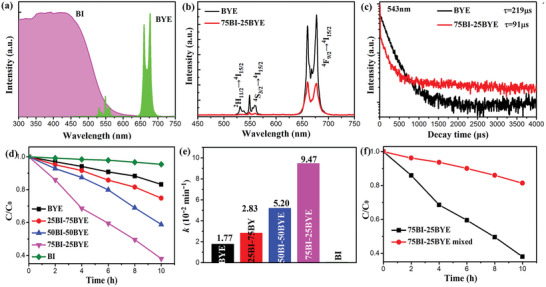
a) Absorption of BI and UC emission spectral of BYE; b) UC emission spectra of BYE and 75BI‐25BYE and c) lifetime decay curves of 543 nm under 980 nm excitation; d) photocatalytic degradation of BPA, e) apparent rate constants of BI‐BYE heterojunctions and f) photocatalytic degradation of BPA of 75BI‐25BYE physical mixture under NIR light illumination.

In addition to light absorption ability and photogenerated carrier separation efficiency, UC emission also contributes to enhanced photocatalytic activity. The BI absorption spectrum plotted with the BYE UC emission spectrum reveals a significant overlap in the green region (**Figure** **6**a), which is a prerequisite for energy transfer. To verify that the BI‐BYE heterojunction made use of UC luminescence, a 980 nm laser was used to obtain the UC luminescence spectra of BYE and 75BI‐25BYE (Figure 6b and Figure S14, Supporting Information). The emission peaks observed at 525 nm, 543 nm, and 672 nm in the visible light region were respectively assigned to the Er^3+^ ion H_11/2_ → ^4^I_15/2_, ^4^S_3/2_ → ^4^I_15/2_, and ^4^F_9/2_ → ^4^I_15/2_ transitions, respectively. Furthermore, the UC luminescence intensity of 75BI‐25BYE was significantly lower than that of BYE, demonstrating an effective energy transfer from BYE to BI. To further demonstrate the energy transfer process between BYE and BI, the fluorescence lifetimes of the Er^3+^ levels ^4^S_3/2_ and ^4^F_9/2_ were measured and fitted by biexponential function (Figure 6c and Figure S15, Supporting Information). It was clearly observed that the lifetime of Er^3+^ at the ^4^S_3/2_ level was significantly lower for 75BI‐25BYE than for BYE, while the lifetime of Er^3+^ at the ^4^F_9/2_ level showed no significant difference. The accelerated decay lifetimes of 75BI‐25BYE indicate the presence of a highly efficient Förster resonance energy transfer (FRET) from Er^3+^ at the ^4^S_3/2_ level to BI.^[^ ^13^, ^65^ ^]^ This highly efficient FRET process implies that this heterojunction can utilize green light for photocatalytic reactions, which may improve the utilization efficiency of NIR light and the overall photocatalytic performance.

The significance of UC luminescence to the photocatalytic process was further studied by the photocatalytic degradation of BPA under NIR light irradiation. As shown in Figure 6d, BI had almost no photocatalytic activity because the NIR photon energy was much smaller than its bandgap energy. In contrast, BYE exhibited weak photocatalytic activity for BPA degradation, which was attributed to the utilization of NIR light via Yb^3+^/Er^3+^ UC emission. However, this was still lower than the photocatalytic activity of the BI‐BYE heterojunctions. The 75BI‐25BYE heterojunction exhibited the highest photocatalytic activity toward BPA degradation, with a degradation rate of up to 62%. The reaction rate of the 75BI‐25BYE heterojunction (*k* = 0.045 h^−1^) was approximately 5.3 times higher than that of BYE (*k* = 0.045 h^−1^) (Figure 6e). This suggested that the UC effect had a crucial role in enhancing the NIR photocatalytic activity of 75BI‐25BYE. In addition, compared with the BI‐BYE heterojunctions, the NIR photocatalytic activity of a mixture of BI and BYE was markedly lower (Figure 6f). The less than 10 nm distance between the BYE and BI in these heterojunctions led to intimate interfacial contact, which promoted the FRET process and improved the photocatalytic efficiency. In contrast, due to the inefficient energy transfer between BYE and BI in a physical mixture, the NIR photocatalytic activity of the mixture of BI and BYE was lower than that of the BI‐BYE heterojunctions. Therefore, this analysis shows that the FRET process is an important factor in the photocatalytic performance of the BI‐BYE heterojunctions and that it is conducive to enhancing the utilization of UC luminescence and the NIR photocatalytic activity.

### 2.4 DFT Calculations

**Figure 7:**
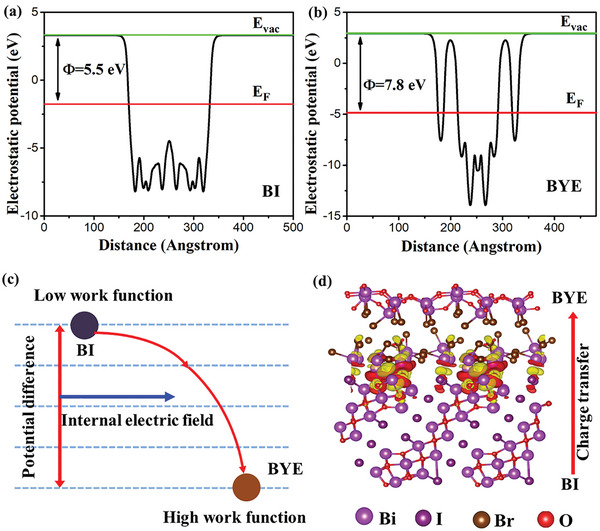
DFT calculated electrostatic potentials of a) BI and b) BYE; c) schematic diagrams of charge transfer driven by potential difference and the formation of internal electric field; d) charge density distribution of BI‐BYE heterojunctions, the yellow and red color represents the electron accumulation and depletion region.

To investigate the interfacial charge transfer path and formation mechanism of the BI‐BYE heterojunction, Density functional theory (DFT) calculations were performed. The work function (*Φ*) is an important interfacial charge transfer parameter, and the *Φ* values were calculated according to the equation: *Φ* = *E*_vac_ − *E*_F,_^[^ ^50^ ^]^ where *E*_vac_ is the potential of vacuum energy and E_F_ is the Fermi energy. The *Φ* value of BI was 5.5 eV and that of BYE was 7.80 eV (**Figure** **7**a,b). BI exhibited a higher *E*_F_ than BYE, which showed that BI and BYE met the theoretical conditions for Z‐scheme heterojunction formation. In this heterojunction, BI and BYE are in close contact, and electrons will spontaneously transfer from BI to BYE until the equilibrium of *E*_F_ is reached, which is consistent with the XPS results, indicating that charge transfer occurred across the BI and BYE interface. As a result, the BI‐BYE heterojunction displays the *Φ* = 6.88 eV (Figure S16, Supporting Information). Subsequently, the electron aggregation region is formed on the BYE side, while a depletion layer exists on the BI side. Thus, an internal electric field (IEF) in the direction of BI to BYE is generated (Figure 7c). The charge density distribution of BI‐BYE was simulated (Figure 7d and Figure S17, Supporting Information) to more intuitively reflect the BI‐BYE heterojunction charge transfer process.^[^ ^66^ ^]^ Yellow in Figure 7d represents the electron accumulation region, while red represents the electron depletion region. It was observed that the BI surface was predominantly covered by red, while BYE was mainly yellow. This suggested the transfer of electrons from BI to BYE via their intimate heterostructure interface. This result was in good agreement with the work function analysis and theoretically demonstrates the formation of the BI‐BYE Z‐scheme heterojunction.

**Figure 8:**
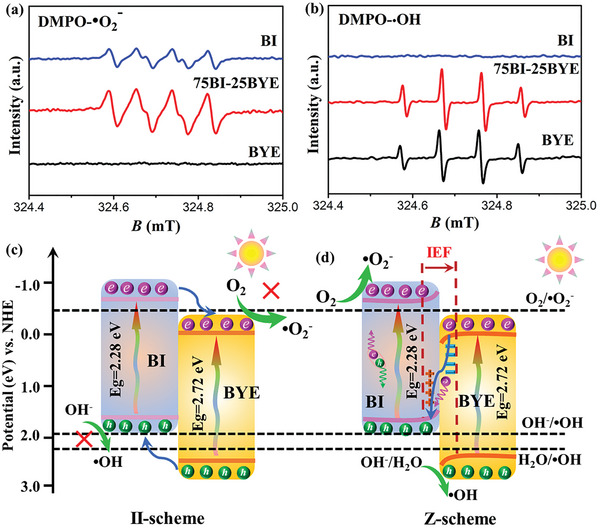
EPR spectra of the a) DMPO‐•O_2_^−^ and b) DMPO‐•OH for BI‐BYE heterojunctions under full‐spectrum light irradiation; c,d) photogenerated electron and hole pair separation and possible reaction mechanistic pathway of BI‐BYE heterojunctions.

The Z‐scheme mechanism of the BI‐BYE heterojunction was verified by 5,5‐dimethyl‐1‐pyrroline N‐oxide (DMPO) spin‐trapping EPR measurements (**Figure** **8**a,b). Under full‐spectrum light irradiation, typical DMPO‐•O_2_^−^ signals were observed for BI and 75BI‐25BYE, whereas no obvious signals were detected for BYE. These results showed that BI and 75BI‐25BYE were favorable for producing •O_2_^−^, because the *E*_CB_ of BI (−0.54 eV) was more negative than that of O_2_/•O_2_^−^ (−0.33 eV versus NHE)^[^ ^67^ ^]^ and met the conditions for generating •O_2_^−^; while the *E*_CB_ of BYE (−0.06 eV) was more positive than that of O_2_/•O_2_^−^. On the other hand, both BYE and 75BI‐25BYE exhibited the characteristic signal of DMPO‐•OH, whereas there was no obvious response in BI. This was because the *E*_VB_ of BYE (2.66 eV) was more positive than that of H_2_O/•OH (2.27 eV versus NHE) and OH^−^/•OH (1.99 eV versus NHE),^[^ ^68^ ^]^ meaning that the generation of •OH was favored; while the *E*_VB_ of BI (1.74 eV) was far negative than that of H_2_O/•OH and OH^−^/•OH. If the BI‐BYE heterojunction was a type‐II heterojunction structure (Figure 8c), the electrons of BI would shift to BYE, which does not have the ability to generate •O_2_^−^. Therefore, the possibility of a type‐II heterojunction was ruled out, further confirming the Z‐scheme charge transfer route for the BI‐BYE heterojunction. More importantly, both the DMPO‐•O_2_^−^ and DMPO‐•OH signals of the 75BI‐25BYE heterojunction were much stronger than those of BI and BYE, showing that the 75BI‐25BYE heterojunction exhibited both strong oxidation and reduction properties for generating abundant •O_2_^−^ and •OH. In addition, the existence OVs can capture electrons and simultaneously adsorb more O_2_ molecules, thus enhancing the formation of •O_2_^−.[^ ^29^ ^]^ These results demonstrate that the electrons tended to remain in the CB of BI and the holes tended to remain in the VB of BYE. Therefore, the EPR results and DFT calculations strongly validate the Z‐scheme charge transfer route of the photogenerated electrons and holes in the BI‐BYE heterojunction.

This Z‐scheme charge transfer pathway between BI and BYE is proposed as shown in Figure 8d. When the BI and BYE nanosheets come into close contact, electrons spontaneously transfer from BI to BYE until the equilibrium of *E*_F_ is reached. When this charge transfer occurs, the energy band edge of BI bends upward, which is due to the loss of electrons. Meanwhile, the energy band edge of BYE bends downward because BYE is enriched in electrons. Under light irradiation, both BI and BYE are stimulated, leading to the production of photogenerated electrons and holes. The IEF accelerates the migration of these photogenerated electrons from the CB of BYE to the VB of BI, where they recombine with the photoinduced holes. Consequently, the BI‐BYE Z‐scheme heterostructure enables the efficient separation of photogenerated electrons and holes. This means that the strong reducibility of the electrons in the *E*_CB_ of Bi_4_O_5_I_2_ and the oxidizability of the holes in the *E*_VB_ of BiOBr are preserved, leading to the production of abundant reactive species. Therefore, the photocatalytic performance of the heterojunction is significantly boosted.

### 2.5 Photocatalytic Mechanism

**Figure 9:**
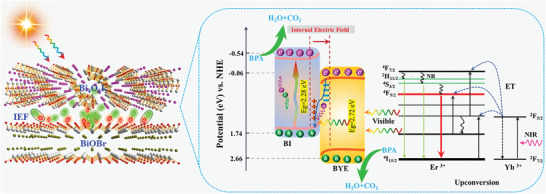
Schematic illustration of the photocatalysis mechanisms for BPA degradation over the BI‐BYE heterojunctions under full‐spectrum light irradiation.

Based on the experimental results and theoretical calculations reported in this work, the potential photocatalytic mechanisms of the BI‐BYE heterojunction are illustrated in **Figure** **9**. Under the UV and visible light irradiation, electron–hole pairs are simultaneously generated at both BYE and BI. The photogenerated electrons in the VB of BYE and BI are excited to their CB, while holes are left in the VB. Due to the IEF across the contact interface between BI and BYE, the photogenerated electrons on the CB of BYE will combine with the holes on the VB of BI via a Z‐scheme charge transfer process. Thus, electrons accumulate in the CB of BI and holes accumulate in the VB of BYE. Subsequently, many of the holes enriched on BYE will oxidize H_2_O and •OH, leading to the production of •OH. The electrons on BI react with O_2_ to generate •O_2_^−^. Hence, this Z‐scheme charge transfer mechanism effectively accelerates photogenerated charge carrier separation and leads to maximized redox ability. This significantly improves the photocatalytic performance of the heterojunction. In addition, the UC materials absorb the NIR light and convert it into visible light photons. These light photons are captured by the BYE and BI due to their matching band gap with the wavelength of the emitted visible light. Under the illumination of NIR light, the Yb^3+^ ions in BYE are excited to the excited state ^2^F_5/2_ from the ground state ^2^F_7/2_. The excited Yb^3+^ can populate the higher energy levels of Er^3+^ ions via the energy transfer process. Green and red emissions centered at 525, 543, and 672 nm occur, and these are attributed to the H_11/2_/^4^S_3/2_ → ^4^I_15/2_ and ^4^F_9/2_ → ^4^I_15/2_ transitions of Er^3+^. Next, the energy of the H_11/2_/^4^S_3/2_ levels directly transfers to the BI via FRET process. Meanwhile, the partly green emissions also can be absorbed by BYE, which is consistent with our previous research results.^[^ ^28^, ^30^ ^]^ The absorption of the UC high‐energy emissions can induce excitations across the band gap, generating electron–hole pairs that lead to further photodegradation. The overall photocatalytic degradation process using full‐spectrum light irradiation can be described as follows:

(3) $$\mathrm{BYE}+h\nu \left(\mathrm{NIR}\right)\to h\nu \left(\mathrm{Vis}\right)\left(\mathrm{UC}\right)$$

(4) $$\mathrm{BYE}+h\nu \left(\mathrm{UV}\right)+h\nu \left(\mathrm{Vis}\right)\to e^{-}+h^{+}$$

(5) $$\mathrm{BI}+h\nu \left(\mathrm{UV}\right)+h\nu \left(\mathrm{Vis}\right)\to e^{-}+h^{+}$$

(6) $$\mathrm{BYE}+\mathrm{BI}+h\nu \left(\mathrm{Vis},\;\mathrm{UC}\right)\to e^{-}+h^{+}$$

(7) $$O_{2}+e^{-}\to \cdot O_{2}^{-}$$

(8) $$H_{2}O/{\mathrm{OH}}^{-}+h^{+}\to •\mathrm{OH}$$

(9) $$•O_{2}^{-},\;•\mathrm{OH},h^{+}+\mathrm{BPA}\to {\mathrm{CO}}_{2}+H_{2}O$$

Based on the above discussion, the enhanced photocatalytic performance of BI‐BYE heterojunction can be ascribed to the synergistic effects of the formed 2D–2D Z‐scheme heterojunction and the UC luminescence of Er^3+^, which boosts the separation of electron–hole pairs and achieves a broadband response under UV‐Vis‐NIR light irradiation.

## 3 Conclusion

In summary, a 2D–2D BI‐BYE Z‐scheme heterojunction was rationally designed and fabricated to take advantage of the UC effect and Z‐scheme heterojunction charge transfer process. Benefiting from the close 2D–2D heterointerface, extremely short charge transfer distances and enhanced FRET efficiency were achieved. The Z‐scheme charge transfer process effectively improved the separation of photogenerated charges and maintained a robust redox capacity, which was supported by XPS, EPR, and DFT analysis. These factors cooperatively contributed to the light harvesting capability and charge separation efficiency of the BI‐BYE heterojunction and also meant that a robust redox capacity was maintained during the photocatalytic process. This led to significantly enhanced photocatalytic performance for BPA degradation. As a result, the optimal 75BI‐25BYE heterojunction demonstrated the best photocatalytic performance, with a BPA degradation rate of 100% achieved within 30 min under full‐spectrum light irradiation. The catalytic activity of 75BI‐25BYE was approximately 6.0 times that of BYE and 2.0 times that of BYE. This work offers a new strategy for developing novel and high‐efficiency UC‐based Z‐scheme catalysts capable of harvesting a broad range of the solar spectrum.

## 4 Experimental Section

### Synthesis of BiOBr:Yb^3+^,Er^3+^ (BYE) Nanosheets

BiOBr:10%Yb^3+^,1%Er^3+^ nanosheets were synthesized by the solvothermal method. First, 5 mmol Bi(NO_3_)_3_·5H_2_O, 1.0 mL Yb(NO_3_)_3_ (0.5 mol L^−1^), 0.5  mL Er(NO_3_)_3_ (0.1 mol L^−1^) were dissolved into 30 mL mannitol solution (0.2 mol L^−1^) and then stirred for 30 min. Second, 2.0 mL KBr solution (2.5 mol L^−1^) was slowly added to the above reaction solution under continuous stirring. After another 10 min of agitation, the mixture was transferred into a Teflon‐lined stainless‐steel autoclave of 50 mL capacity, and the autoclave was then placed in an oven with a temperature of 160 °C for 12 h. Naturally cooled to room temperature, the precipitates were washed with absolute alcohol and deionized water for several times and dried at 70 °C in the air. Finally, the precursor was calcined at 450 °C for 1 h in air with a heating rate of 5 °C min^−1^, which is denoted as BYE.

### Synthesis of Bi_4_O_5_I_2_ (BI) Nanosheets

Bi_4_O_5_I_2_ nanosheets were synthesized by the solvothermal method. First, 5 mmol Bi(NO_3_)_3_·5H_2_O was dissolved into 30 mL mannitol solution (0.1 mol L^−1^) and then stirred for 30 min. Second, 2.0 mL KI solution (2.5 mol L^−1^) was slowly added to the above reaction solution under continuous stirring. After another 10 min of agitation, the mixture was transferred into a Teflon‐lined stainless‐steel autoclave of 50 mL capacity, and the autoclave was then placed in an oven with a temperature of 160 °C for 12 h. Naturally cooled to room temperature, the precipitates were washed with absolute alcohol and deionized water for several times and dried at 70 °C in the air. Finally, the precursor was calcined at 400 °C for 3 h in air with a heating rate of 5 °C min^−1^, which is denoted as BI.

### Synthesis of Bi_4_O_5_I_2_/BiOBr:Yb^3+^,Er^3+^ (BI‐BYE) Z‐Scheme Heterojunctions

The 2D–2D Bi_4_O_5_I_2_/BiOBr:Yb^3+^,Er^3+^ Z‐scheme heterojunctions were prepared by a two‐step solvothermal method. Typically, a certain mass of as‐synthesized BYE and BI were each dispersed in 15 mL of ethanol for 1 h under ultrasonication. Then, the BYE and BI suspensions were mixed together and stirred for 1 h. The mixture was transferred into a 50 mL Teflon‐lined autoclave and heated at 160 °C for 12 h. Naturally cooled to room temperature, the precipitates were washed with absolute alcohol and deionized water for several times and dried at 70°C in air. The prepared samples were denoted as (100−*x*) Bi_4_O_5_I_2_−*x*BiOBr:Yb^3+^,Er^3+^, where *x* refers to the percentage of BiOBr:Yb^3+^,Er^3+^ (*x* = 0, 25, 50, 75, 100), which could be abbreviated as BI, 75BI‐25BYE, 50BI‐50BYE, 25BI‐75BYE, BYE, respectively.

### Characterization

The detailed characterization methods are provided in Supporting Information.

### Photocatalytic Activity Experiment

Photocatalytic activity of samples for BPA decomposition was estimated under Vis light (400 nm < *λ*< 760 nm), NIR light (760 nm < *λ*< 1100 nm), full‐spectrum light (300 nm < *λ*< 1100 nm) with a 500 W Xe lamp (CEL‐LAX500) as the irradiation source. In a typical process, 20 mg sample was added into the BPA solutions (40 mL, 10 mg L^−1^), and stirred for 2 h in the darkness to ensure absorption–desorption equilibrium. 3 mL reaction solution was taken out at regular times. The supernatant solutions were analyzed by UV‐1800 spectrophotometer.

## Conflict of Interest

The authors declare no conflict of interest.

## Supporting information

Supporting Information

## Data Availability

The data that support the findings of this study are available from the corresponding author upon reasonable request.
