# Supplementary material for: Enhancement of Charge Separation and NIR Light Harvesting through Construction of 2D–2D Bi4O5I2/BiOBr:Yb3+, Er3+ Z‐Scheme Heterojunctions for Improved Full‐Spectrum Photocatalytic Performance
Source: Adv Sci (Weinh). 2023 Feb 21;10(13):2207514. doi: 10.1002/advs.202207514 (PMC10161072; doi:10.1002/advs.202207514)
Supplement: Supplementary file 1 — Supporting Information [file ADVS-10-2207514-s001.pdf]

Supporting Information

Enhancement of charge separation and NIR light harvesting through construction of 2D-2D $Bi_4O_5I_2$/BiOBr:Yb^{3+},Er^{3+} Z-scheme heterojunctions for improved full-spectrum photocatalytic performance

Yongjin Li[1*], Junhao Ma[1], Liang Xu[1], Tong Liu[2], Taizhong Xiao[1], Daomei Chen[3*], Zhiguo Song[1], Jianbei Qiu[1], Yueli Zhang[2*]

[1] School of Materials Science and Engineering, Kunming University of Science and Technology, Kunming, 650093, China

[2] State Key Laboratory of Optoelectronic Materials and Technologies, School of Materials Science and Engineering, Sun Yat-Sen University, Guangzhou, 510275, China

[3] National Center for International Research on Photoelectric and Energy Materials, School of Materials and Energy, Yunnan University, Kunming 650091, China

[*]Corresponding Author

E-mail addresses: liyongjin@kust.edu.cn (Y. Li); stszyl@mail.sysu.edu.cn (Y. Zhang); dmchen@ynu.edu.cn (D. Chen)

Experimental

Characterization

The crystal structures of the samples were characterized by X-ray diffraction (XRD) using a Rigaku Smartlab powder diffractometer with Cu Ka radiation. The morphologies of the samples were studied using scanning electron microscopy (SEM, Gemini 500) and transmission electron microscopy (FEI Tecnai G2 F30 and G2 Spirit). The elemental analysis was performed by X-ray photoelectron spectroscopy (XPS, ESCA Lab 250, Al Ka radiation). The The UV–Vis–NIR absorption spectra of samples were obtained by a spectrophotometer (Shimadzu, UV3600), using the $BaSO_4$ as the reflectance sample. Photoluminescence spectra and decay profiles were recorded using an Edinburgh Instruments FLS980 spectrometer equipped with a 980 nm diode laser as the excitation source, and R928P and R5509-72 photomultiplier as detectors. In addition, photocurrent analysis and electrochemical impedance spectroscopy (EIS) were conducted using an Electrochemical Workstation (CHI760E, Shanghai Chenhua, China) with a standard three-electrode system. The signal of active radicals in the degradation process is measured by Electron paramagnetic resonance (EPR, Bruker A300). Total organic carbon (TOC) was analyzed on a TOC analyzer (Shimadzu, Japan) to evaluate the degree of mineralization of reaction solution. A liquid chromatography two stage tandem mass spectrometry (LC-MS) (Thermo Fisher Scientific Inc., USA) was used to determine the intermediate products from BPA degradation.

Detection of reactive species

Electron paramagnetic resonance (EPR) signals of reactive species were obtained with a Bruker A300 using 5, 5-dimethyl-1-pyrroline N-oxide (DMPO) as the spin-trap reagent.

For investigating the active species that are responsible for the photocatalytic activity, severer scavengers, including 1 mmol L^{-1} 1,4-benzoquinone (BQ, $\bullet O_2^-$ scavenger), 1 mmol L^{-1} ethylenediaminetetraacetic acid disodium salt dihydrate (EDTA-2Na, h^+ scavenger), and 10 mmol L^{-1} tertiary butanol (*t*-BuOH, •OH scavenger), were individually added into the 40 mL BPA solution. The testing was similar to that in the above photocatalytic experiment.

Theoretical calculations

All calculations were performed with the density functional theory (DFT) implemented in VASP, applying a generalized gradient correlation functional, along with the generalized gradient approximation (GGA) exchange and correlation functions in the scheme of Perdew-Burke-Ernzerh of (PBE). The band structure and DOS plots of BI and BYE were obtained by using the first-principles DFT calculations to specify the electronic structure in photocatalyst. The valence electronic configurations are the states of Bi 6p, O 2p, Br 4p and I 5p for the ground-state electronic structure calculations. A plane wave cutoff energy of 520 eV was used in the calculations. The Brillouin zone was sampled with a 2 × 4 × 2, 5 × 5 × 3 and 2 × 2 × 1 Monkhorst Pack grid of BI and BYE and heterojunction. All the atoms of the BI and BYE and heterojunction are fully relaxed to their equilibrium positions with an

energy convergence of 1×10^{-5} eV while the force applied on each atom is less than 0.01 eV/Å.

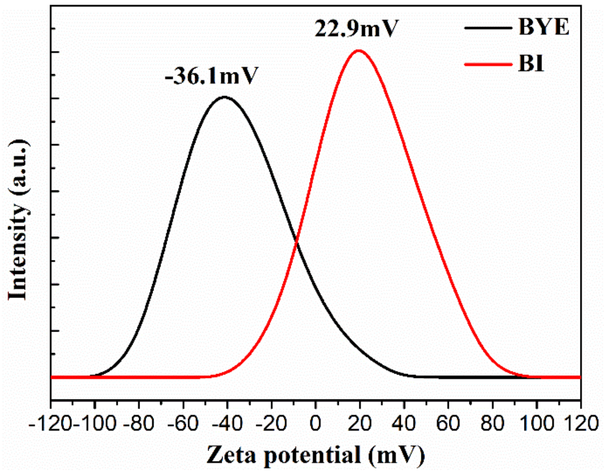

Fig. S1. Zeta potentials of BYE and BI in ethanol.

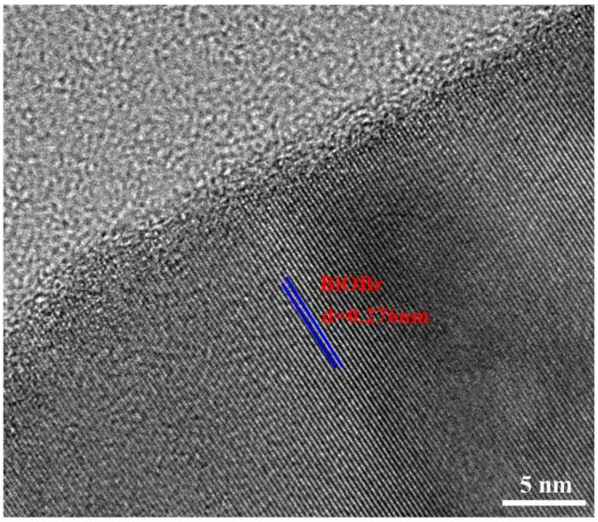

Fig. S2. HRTEM image of pure BiOBr.

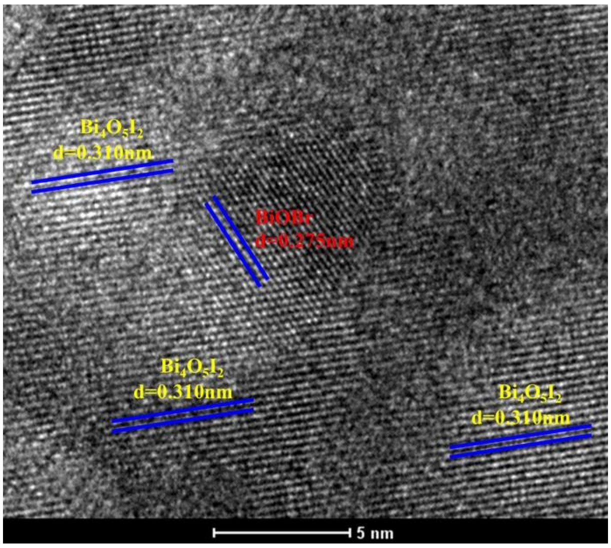

Fig. S3. HRTEM image of 75BI-25BYE.

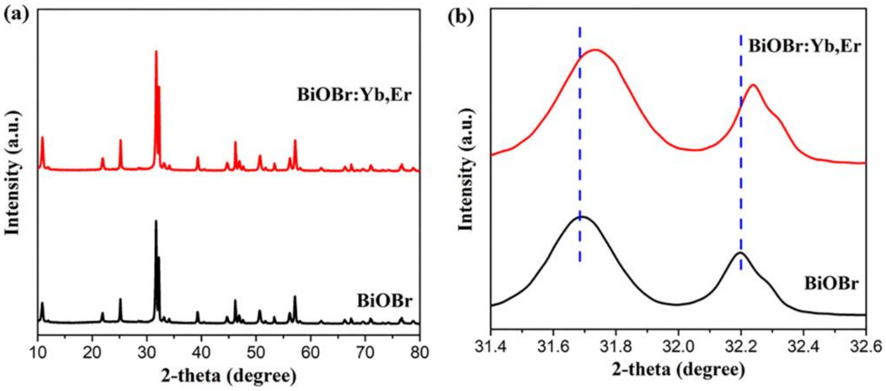

Fig. S4. (a) XRD patterns of pure BiOBr and BiOBr: 10%Yb^{3+}/1%Er^{3+} (BYE) nanosheets; (b) The main diffraction peak near 2θ = 31.4º - 32.6º of sample.

In order to explain the doping style of Yb^{3+} and Er^{3+} ions, the XRD patterns of pure BiOBr and BiOBr: 10%Yb^{3+}/1%Er^{3+} (BYE) nanosheets were carried out, as shown in Fig. S1. It was found that all diffraction peaks of the products were well indexed to the tetragonal phase BiOBr (JCPDS No. 73-2061). And no other diffraction peaks were observed, indicating the successful incorporation of Yb^{3+}, Er^{3+} into the BiOBr host. To reveal the subtle differences caused by Yb^{3+} and Er^{3+} ions doping, a selected region of diffraction peaks in the range of 31.4º - 32.6º was studied,

as shown in Fig. S1. Compared with the pure BiOBr, the diffraction peaks showed a slight shift to a high angle, which indicate that Yb^{3+} and Er^{3+} ions can be doped into the host lattice through the substitution. This should be attributed the substitution of lager Bi^{3+} ion (1.03 Å) by smaller Er^{3+} (0.89 Å) and Yb^{3+} (0.87 Å) ions, which can induce the shrinking of the host lattice.

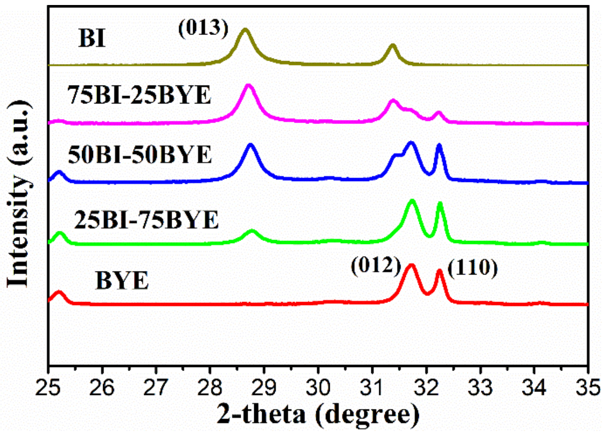

Fig. S5. The main diffraction peak near 2θ = 25º~35º of BI-BYE heterojunctions.

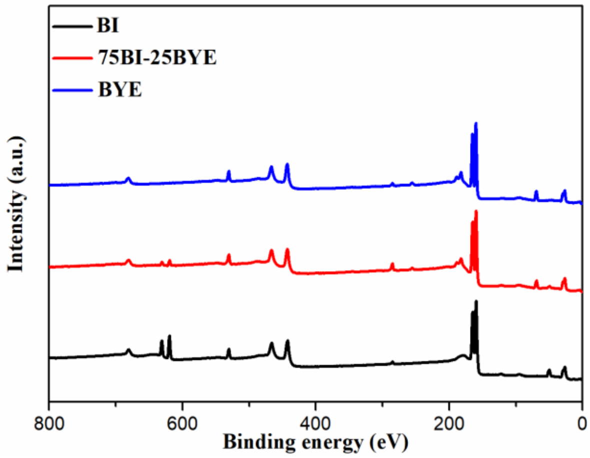

Fig. S6. XPS full spectra of the BI-BYE heterojunctions.

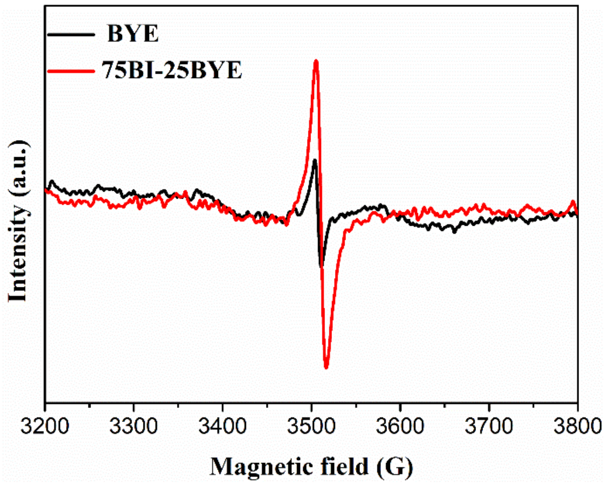

Fig. S7. EPR spectra of the BI-BYE heterojunctions.

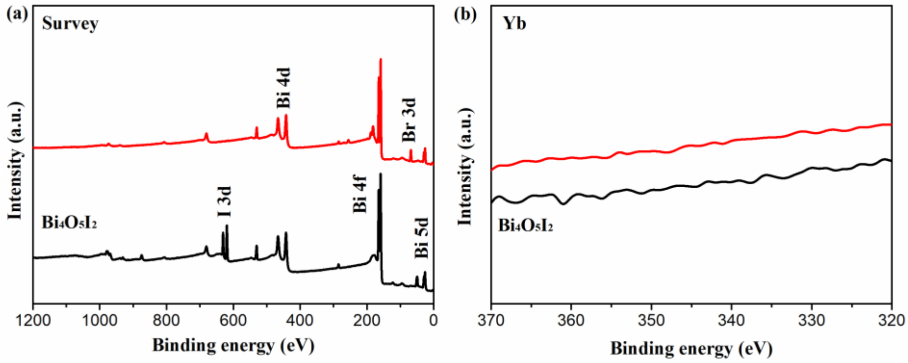

Fig. S8. XPS spectrum of pure $Bi_4O_5I_2$ and BiOBr. (a) Survey spectra; (b) High-resolution XPS spectra of Yb 4p.

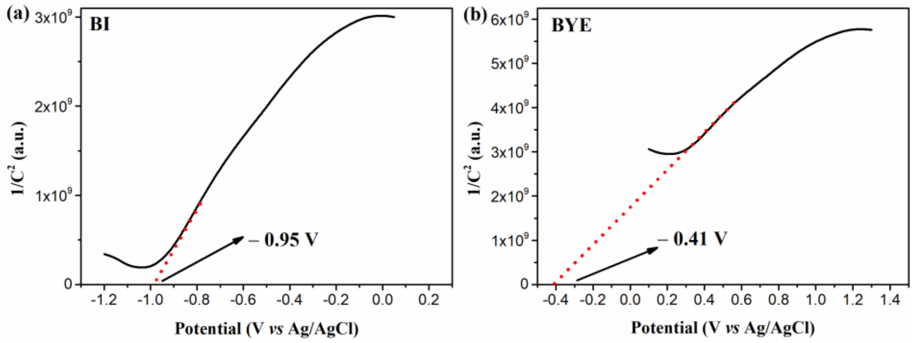

Fig. S9. Mott-Schottky plots of BI and BYE.

Mott-Schottky (M−S) plots were used to elucidate the conduction type and flat band potentials (E_{fb}) of BI and BYE. As shown in Fig. S6, the M−S slopes of both BI and BYE exhibits a positive slope, indicating that both samples are n-type semiconductors. In addition, the E_{fb} of BI and BYE were −0.95 V and −0.41 V *vs.* Ag/AgCl, respectively. The E_{fb} value could be further converted into a normal hydrogen electrode (NHE) potential Equation as follows [1].

$$E_{fb\ (vs.\ NHE)} = E_{fb\ (pH=0\ vs.\ Ag/AgCl)} + E_{AgCl} + 0.059 \times pH \qquad (S1)$$

where the pH value is around 6.5, and E_{AgCl} is 0.197 V. Consequently, the E_{fb} of BI and BYE were −0.37 eV and 0.17 eV *vs.* NHE, respectively. Generally, the E_{fb} is more positive by approximately 0.20 eV than the conduction band potential (E_{CB}) for an n-type semiconductor [2,3]. Therefore, the E_{CB} of BI and BYE were estimated to be −0.57 and – 0.03 eV vs. NHE, then the valence band potentials (E_{VB}) were calculated to be 1.71 and 2.69 eV, respectively.

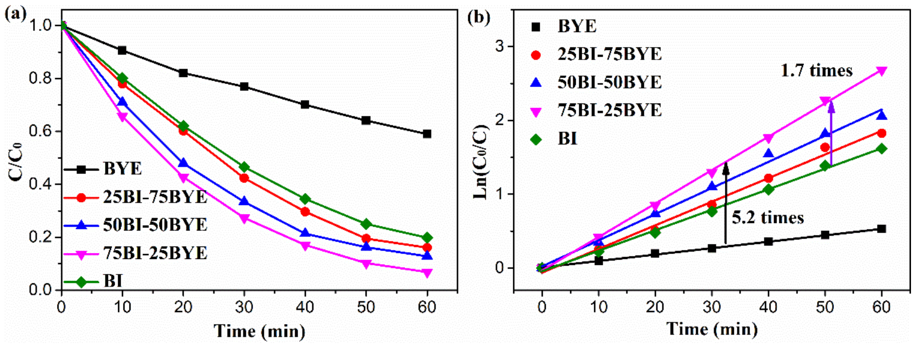

Fig. S10. (a) Photocatalytic degradation of BPA and (b) apparent rate constants of BI-BYE heterojunctions under visible light irradiation.

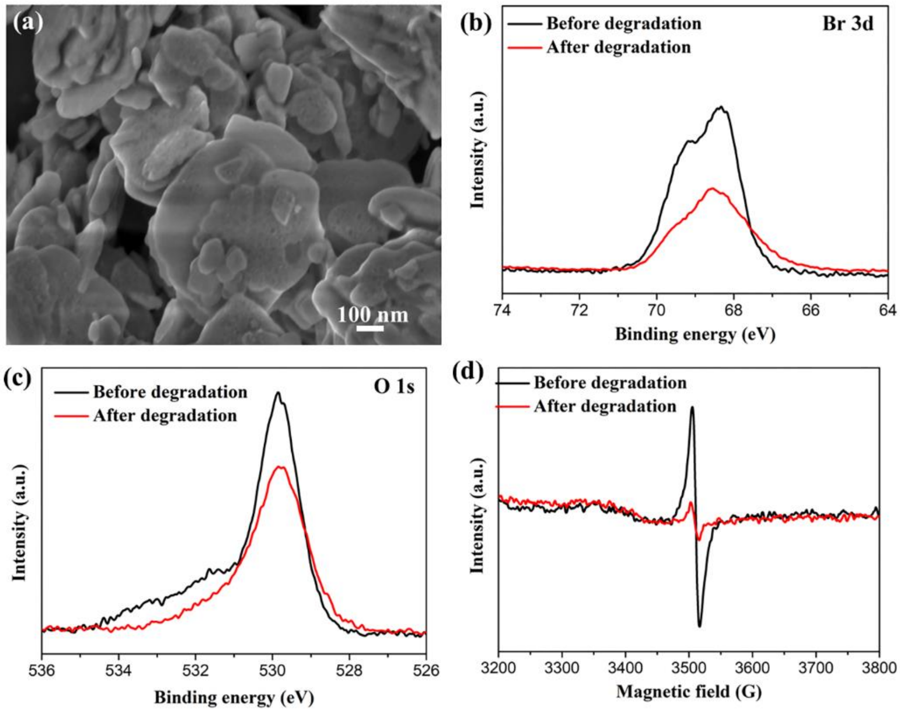

Fig. S11. (a) SEM image, XPS spectra of (b) Br 3d and (c) O 1s; (d) EPR spectra of 75BI-25BYE before and after 5 cycles reaction.

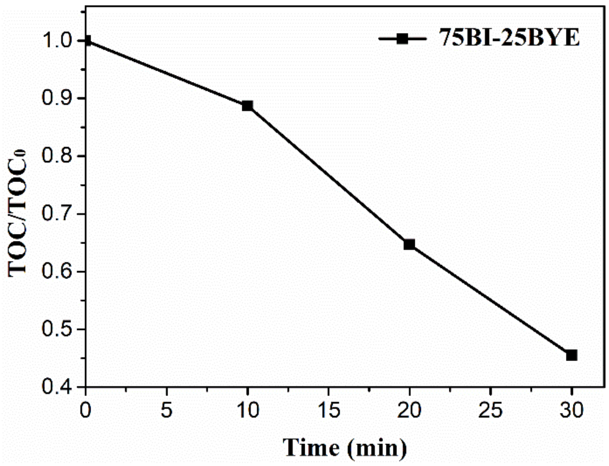

Fig. S12. TOC removal curves of 75BI-25BYE under full-spectrum light irradiation.

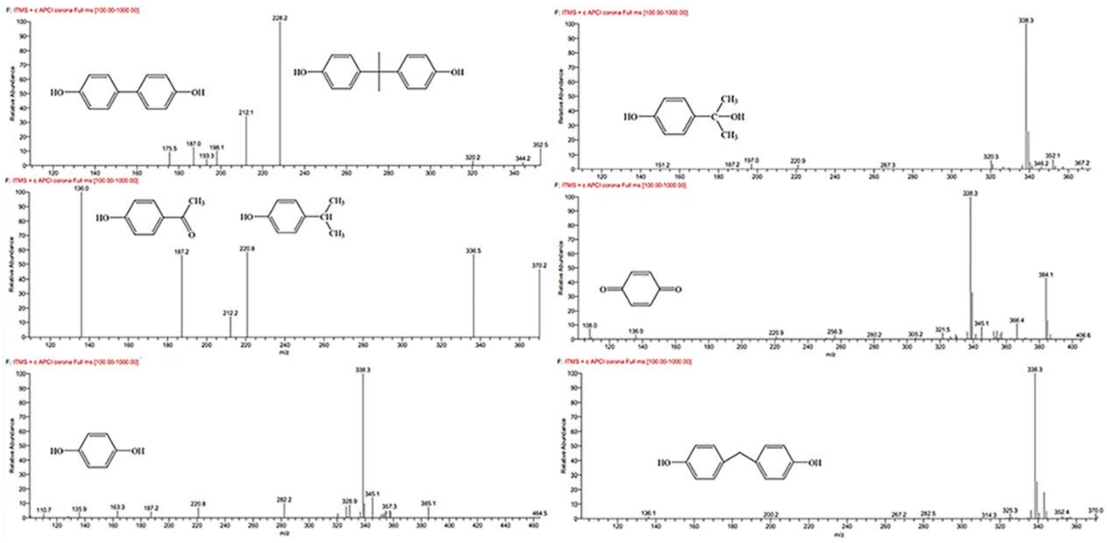

Fig. S13. MS spectra of the intermediates from BPA degradation over 75BI-25BYE heterojunction under full-spectrum light irradiation.

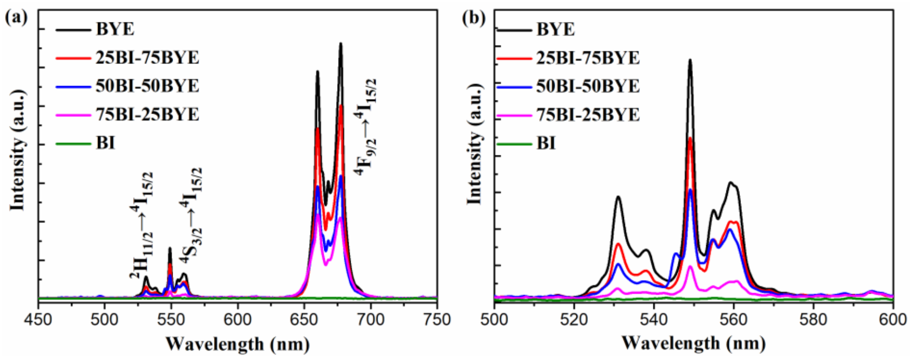

Fig. S14. (a) UC luminescence spectra and (b) green emission of BI-BYE heterojunctions.

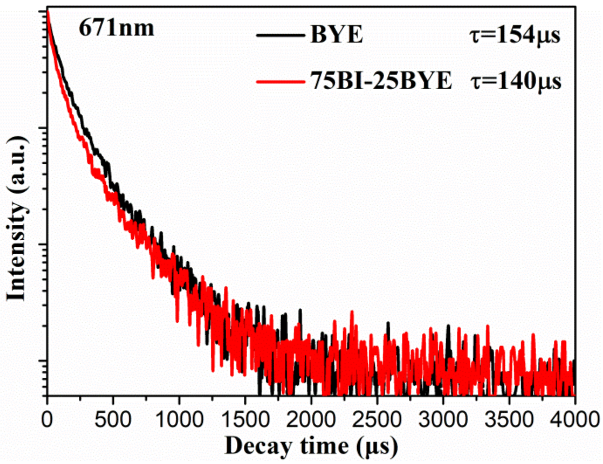

Fig. S15. Lifetime decay curves of 671 nm under 980 nm excitation.

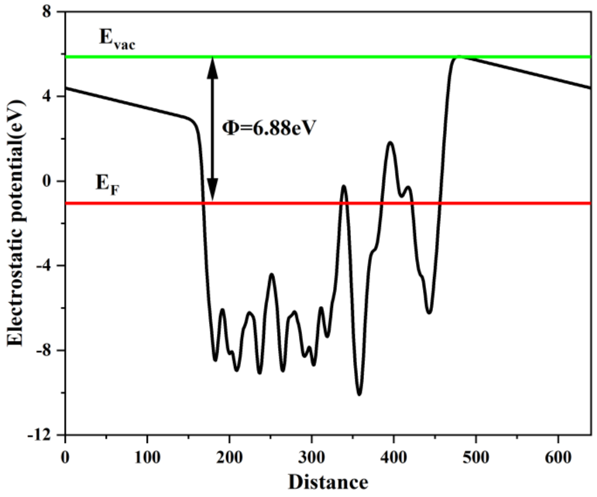

Fig. S16. DFT calculated electrostatic potentials of BI-BYE heterojunctions.

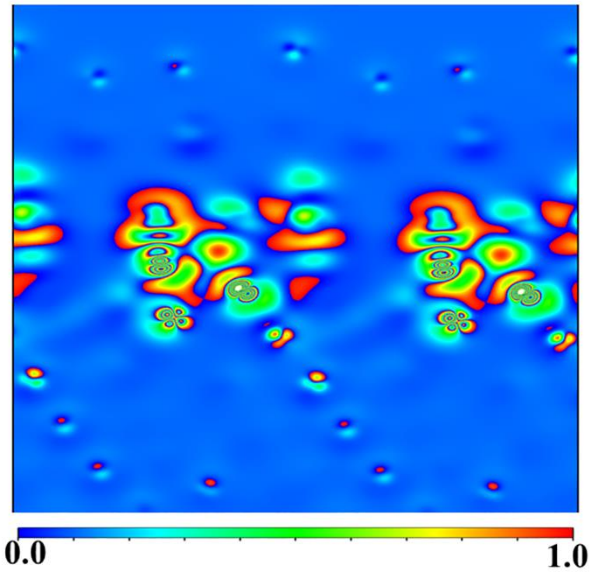

Fig. S17. Electronic location function (ELF) of BI-BYE heterojunctions.

Table S1. Degradation intermediates of BPA analyzed by LC-MS.

No.	Compound	Structure	Formula	m/z
P0	Bisphenol A	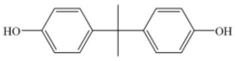	$C_{15}H_{16}O_2$	228
P1	4,4'-methylenediphenol	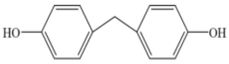	$C_{13}H_{12}O_2$	200
P2	Hydroquinone	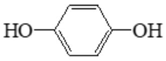	$C_6H_6O_2$	110
P3	4-benzoquinone	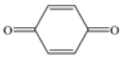	$C_6H_4O_2$	108
P4	4-isopropylphenol	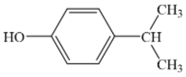	$C_9H_{12}O$	135
P5	4-isopropanolphenol	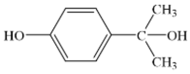	$C_9H_{12}O_2$	151
P6	4-hydroxyacetophenone	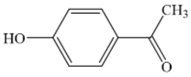	$C_8H_8O_2$	136
P7	4,4'-dihydroxybiphenyl	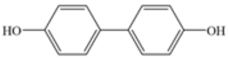	$C_{12}H_{10}O_2$	187

Table S2 Resistance fitting values of different samples according to the Nyquist polts.

Samples	Rs (KΩ)	Rct (KΩ)	CPE-P	CPE-T
BYE	0.107	875.8	0.809	0.096
75BI-25BYE	0.085	31.76	0.708	0.128
BI	0.093	587.4	0.740	0.078

Rct: charge transfer resistance;

Rs: electrolyte solution resistance;

CPE: constant phase element.

References

[1] I. S. Kwon, I. H. Kwak, T. T. Debela, H. G. Abbas, Y. C. Park, J. Ahn, J. Park, H. S. Kang, *ACS Nano* **2020**, *14*, 6295.

[2] W. Yang, K. Sun, J. Wan, Y. Ma, J. Liu, B. Zhu, L. Liu, F. Fu, *Appl. Catal. B: Environ.* **2023**, *320*, 121978.

[3] X. Wang, X. Wang, J. Huang, S. Li, A. Meng, Z. Li, *Nat. Commun.* **2021**, *12*, 4112.
